# Impact of Body Mass Index on Arterial Stiffness in Young Prehypertensives: A Cross Sectional Study

**Published:** 2017-12-27

**Authors:** Velusami Deepika, Raman Vijayakumar

**Affiliations:** ^1^Research scholar in Bharath University, Chennai, India; ^2^Department of Physiology, Sri Manakula Vinayagar Medical College and Hospital, Puducherry, India; ^3^Department of Physiology, Sri Lakshmi Narayana Institute of Medical Sciences and Research, Affliated to Bharath University, Chennai, India

**Keywords:** Body mass index, Prehypertension, Arterial stiffness

## Abstract

**Background:** Obesity and hypertension pose a big threat to public health. Targeting the prehypertension stage to evaluate the vascular damage due to obesity can help us to plan early interventions.

**Study design:** A cross-sectional study.

**Methods:** This study as a part of the medical heath checkup program was conducted by the Medical College in Puducherry, India on all medical students of age group 18-25 years. Blood pressure (BP) recordings and body mass index (BMI) were classified into following four groups: Group1: Normotensives with normal BMI (n=109); Group 2: Normotensives with higher BMI (n=89); Group 3: Prehypertensive subjects with normal BMI (n=50); and Group 4: Prehypertensive subjects with higher BMI (n=99). Arterial stiffness, body fat composition, and lipid profile were evaluated. Correlation of arterial stiffness indices with BMI, BP, body fat, visceral fat, lipid profile were done using Pearson’s correlation and the contribution of BMI to arterial stiffness was assessed using univariate regression analysis.

**Results:** BMI, arterial stiffness, body fat, visceral fat, total cholesterol and total triglyceride showed a significant increase in prehypertensive group with higher BMI as compared to other groups. BMI showed significant correlation with arterial stiffness (*P*<0.0001) and found to be an independent contributing factor for arterial stiffness development in prehypertensives.

**Conclusions:** Vascular damage was seen in the prehypertensive stage itself and was more pronounced in individuals with higher BMI.

## Introduction

With the rapid socioeconomic and nutritional transition in last few decades, obesity and hypertension have become the major cardiovascular (CV) risk factors. Even a mild elevation in blood pressure (BP) (115/75 mmHg) is associated with increased CV risk^1^. Therefore, in 2003, the Joint National Committee on Prevention, Detection, Evaluation, and Treatment of High BP introduced a new term “Prehypertension”, a precursor stage of hypertension, defined as a condition where systolic blood pressure (SBP) ranges from 120 to 139 mmHg, and/or diastolic blood pressure (DBP) ranges from 80 to 89 mmHg^2^. Prehypertension has an increased risk of converting to full-blown hypertension, and increased risk of CV events^3^, if left unaddressed. The incidence of prehypertension in India is increasing at an alarming rate, with the prevalence rate of 55% in South India^4^ and 32.2% in North India^5^, thus posing a potential health threat and economic burden to the modern society. With these perspectives in mind, targeting the vulnerable apparently healthy young individuals for earlier identification of prehypertension is justified and prudent.

Obesity is defined as the overaccumulation of body fat and correlates to a risk of high blood pressure^6^ and persistent elevated BP is reported to be the major determinant of arterial stiffness progression and vascular damage^7^.Arterial stiffness is emerging as an interesting tissue biomarker for cardiovascular risk stratification^8^. It is also proved in animal model that arterial stiffening precedes systolic hypertension in diet-induced obesity^9^. Arterial compliance decreases with increase in adiposity^10^, but literature indicating the contribution of body mass index (BMI) to arterial stiffness in prehypertensive individuals is very meager and conflicting.

The present study was undertaken to assess the nature and magnitude of arterial stiffness and the role of BMI as an independent contributor for arterial stiffness in prehypertensive individuals.

## Methods

### Study design

This cross-sectional study was conducted in Sri Manakula Vinayagar Medical College and Hospital, Madagadipet, Puducherry, India as a part of the medical health check-up program. It was conducted for the students studying Bachelor of Medicine and Bachelor of Surgery (MBBS) course in the Medical College.

The study was initiated after getting approval from the Institute Human Ethical Committee and informed consent was taken from the participants before the study.

### Sample size and sampling

Considering the proportion 32.2%^5^ design effect-1, 95% CI and 10% of non-response, the sample size was calculated as 340 (calculated by Open Epi Software Version). The participants were selected by multistage sampling. From each year (strata), several subjects (clusters) were randomly selected and were enrolled.

### Recruitment of subjects

Overall, 514 students of age group 18-25 yr were enrolled for the health check-up program during Jun 2016-Oct 2016. A detailed review of medical history through structured questionnaire and physical examination were performed to rule out any acute or chronic illness. All baseline characteristics and anthropometrics, height, weight, waist circumference (WC) and Hip circumference (HC) were acquired from all study participants. BMI was calculated using the formula: Weight in kg/ (Height)^2^ in meters. BP was measured in the right arm in the sitting position using a standard mercury sphygmomanometer after a 10-min rest period. Three measurements were taken at 5 min interval and the mean of three measurements was considered for analysis.

Subjects with SBP greater than 130 mmHg and DBP greater than 90 mmHg, with any form of medical illness or any drug treatment were excluded from the study. Based on exclusion criteria, 105 subjects were excluded and 62 subjects were unwilling to participate in the study after listening to the study protocol. Therefore, 347 subjects were enrolled.

### Study groups

Based on the subjects BP recordings as per JNC-7 classification^2^ and BMI as per WHO recommendation on BMI for Asian population ^11^ they were classified into four groups as follows:

**Group1:**Normotensives with normal BMI (n=109): Healthy subjects having systolic BP 100–119 mm Hg, diastolic BP 60–79 mmHg, and BMI 18.5-22 kg/m^2^.

**Group2:** Normotensives with higher BMI (n=89): Healthy subjects having systolic BP 100–119 mm Hg, diastolic BP 60–79 mmHg, and BMI 23 kg/m^2^ or above.

**Group3:** Prehypertensive subjects with normal BMI (n=50): Healthy subjects having systolic BP 120–139 mmHg, diastolic BP 80–89 mmHg, and BMI 18.5-22.9 kg/m^2^.

**Group 4:** Prehypertensive subjects with higher BMI (n=99): Healthy subjects having systolic BP 120–139mmHg, diastolic BP 80–89 mmHg, and BMI 23 kg/m^2^ or above.

### Principle and Calculation of arterial stiffness indices

Though various invasive and noninvasive techniques are available for the assessment of arterial stiffness, the most simple, validated, noninvasive technique that is independent of operator skill, widely used in clinical set up and which is gaining substantial interest in recent years is the pulse trace system, that records the digital volume pulse (DVP)^12^. This technique is based on measuring infrared light transmission through the finger (photoplethysmography) with wavelength of 940 nm. The main principle of this device is the conversion of pressure changes to voltage changes by means of the pressure transducer. It analyses two major measures of vascular function: stiffness index (SI) and reflection index (RI). Similar assessment of arterial stiffness was reported earlier^13,14^.

DVP contains two peaks: systolic peak and diastolic peak. The former peak is due to pulse wave transmitted from the left ventricle to the finger directly and the diastolic peak arises from pulse wave transmitted along the aorta to the small arteries in the lower part of the body, from where they are again reflected along the aorta as a reflected wave. This path length is directly proportional to the subject’s height (h). Pulse transit time (PTT) is the time duration between systolic peak and diastolic peak. Magnitude of systolic and diastolic peak was also measured. Stiffness index and Reflective index were calculated ^13^ by the following formulas:

Stiffness index (SI) = Subject’s height (h)/PTT.

Reflection index (RI) = Magnitude of diastolic peak (b)/ Magnitude of systolic peak (a) × 100

### Protocol for arterial stiffness measurement

Subjects were requested to report to the Physiology lab and DVP was measured in a temperature controlled room (22±2°C), in the right index finger for five min using the Digital Polyrite (RMS vital module LF201308, India). Pulse wave contour analysis was done using the Polyrite D software that gave the PTT, SI, and RI.

### Body composition measurement

After entering age, gender and height taken by stadiometer subject were allowed to stand on the instrument after its calibration. A digital, portable noninvasive instrument Omron KaradaScan ( Model HBF-510, Japan) working on principle of tetrapolar bioelectrical impedance analysis was used that passes electric current of 500 μAmp at frequency 5 kHz to scan the whole body to derive regional body composition.

### Biochemical measurements

Five ml of fasting venous blood sample was collected from the subjects during the medical health check-up program and lipid profile were evaluated. Serum total cholesterol (TC) was measured by cholesterol oxidase method and triglyceride (TG) levels were measured by glycerol kinase- peroxidase method. High-density lipoprotein cholesterol (HDL-c) was measured by divalent cation precipitation method using reagent kits adapted to an automated blood analyzer. Very low-density lipoprotein cholesterol (VLDL-c) was calculated by dividing the triacylglycerol concentration by 5 and low-density lipoprotein cholesterol (LDL-c) by using Friedwald’s equation [TC-(VLDL+HDL)].

### Data analysis

SPSS ver. 13 (SPSS Software Inc., Chicago, IL, USA) was used for statistical analysis. All data were expressed as mean±SD and frequencies. Normality of data was tested by Kolmogorov Smironov test and comparison between groups were done using one way ANOVA and post-hoc by Tukey- Krammer test. The association was assessed by Pearson correlation analysis. The independent contribution of various factors like age, BMI, BHR, SBP and

DBP to arterial stiffness indices was assessed by univariate regression analysis. *P* value of less than 0.05 was considered statistically significant.

## Results

The overall prevalence of prehypertension was found to be 42.94% and the prevalence of individuals with higher BMI was 54.18%. The response rate was reported as 67.5%. The group 4 individuals that are prehypertensives with higher BMI (95% CI; 28.09, 30.01) reported a significant increase (*P* <0.0001) in weight, BMI, WC, HC, SBP, DBP and heart rate (HR) compared to the other groups. (Table 1 and Table 2).

**Table 1:** Comparison of age, anthropometric parameters, blood pressure and arterial stiffness indices between different groups

**Parameters**	**Group 1** **(n=109)**		**Group 2** **(n=89)**		**Group 3** **(n=50)**		**Group 4** **(n=99)**		***P*** **value**
	**Mean**	**SD**	**Mean**	**SD**	**Mean**	**SD**	**Mean**	**SD**	
Age (yr)	19.43	1.25	18.97	1.12	20.57	1.34	20.48	1.29	0.001
Weight (kg)	51.53	7.19	68.45	9.50	55.41	7.17	79.91	18.20	0.001
Waist circumference (cms)	77.28	6.47	88.94	9.04	69.49	14.89	83.94	22.63	0.001
Hip circumference (cm)	89.23	7.01	100.28	7.43	79.69	17.20	95.30	22.66	0.001
Body mass Index (kg/m^2^)	20.00	1.74	25.52	2.20	20.09	1.68	29.05	4.80	0.001
Heart rate (b/min)	75.15	7.03	77.04	6.07	88.14	7.31	88.91	7.97	0.001
Systolic blood pressure (mmHg)	108.42	5.99	110.84	6.11	126.06	3.90	127.46	4.73	0.001
Diastolic blood pressure (mmHg)	67.08	6.23	66.54	6.98	76.46	7.88	75.63	8.50	0.001
Reflection Index (%)	39.02	8.50	43.10	9.03	44.44	13.81	49.28	11.61	0.001
Stiffness Index (m/sec)	4.21	1.16	5.67	1.69	4.50	0.97	6.05	1.61	0.001

Group 1: Normotensives with normal BMI; Group 2: Normotensives with higher BMI; Group 3: Prehypertensives with normal BMI; Group 4: Prehypertensives with higher

**Table 2:** Comparison of various characteristics within groups using one way ANOVA (*P* values)

**Parameters**	**Group** **1 vs 2**	**Group** **1 vs 3**	**Group** **1 vs 4**	**Group** **2 vs 3**	**Group** **2 vs 4**	**Group** **3 vs 4**
Age (yr)	0.040	0.001	0.001	0.001	0.001	0.960
Weight (kg)	0.001	0.170	0.001	0.001	0.001	0.001
Waist circumference (cms)	0.001	0.010	0.010	0.001	0.080	0.001
Hip circumference (cm)	0.010	0.010	0.020	0.001	0.09	0.001
Body mass Index (kg/m^2^)	0.001	0.001	0.001	0.001	0.001	0.001
Heart rate (b/min)	0.240	0.001	0.001	0.001	0.001	0.930
Systolic blood pressure (mmHg)	0.010	0.001	0.001	0.001	0.001	0.440
Diastolic blood pressure (mmHg)	0.950	0.001	0.001	0.001	0.001	0.920
Reflection Index (%)	0.030	0.010	0.001	0.880	0.001	0.040
Stiffness Index (m/sec)	0.001	0.620	0.001	0.001	0.250	0.001
Body Fat (%)	0.001	0.001	0.001	0.001	0.940	0.001
Visceral fat (%)	0.001	0.350	0.001	0.001	0.001	0.001
Subcutaneous fat-whole body (%)	0.010	0.001	0.030	0.001	0.950	0.001
Subcutaneous fat-trunk (%)	0.001	0.001	0.010	0.001	0.990	0.001
Subcutaneous fat-arms (%)	0.280	0.001	1.000	0.001	0.290	0.001
Subcutaneous fat-legs (%)	0.010	0.001	0.390	0.001	0.390	0.001
Total cholesterol (mg/dL)	0.001	0.001	0.001	0.001	0.001	0.001
Triglycerides (mg/dL)	0.001	0.001	0.001	0.001	0.001	0.001
High density lipoprotein cholesterol (mg/dL)	0.001	0.001	0.001	0.001	0.001	0.001
Low density lipoprotein cholesterol (mg/dL)	0.001	0.001	0.001	0.480	0.970	0.710

Reflection index reported a significant increase in all groups when compared to the group 1, normotensives with normal BMI. Group 4 showed significant increase when compared to group 2 and group 3. The higher BMI groups (group 2 and 4) showed significant increase (*P* <0.001) in stiffness index when compared to group 1. Group 3 showed significant increase in SI when compared to group 2 (Table 1).

The body fat showed a significant increase in all groups in comparison to group 1. In addition, body fat reported a significant increase, when group 2 and 3 were compared. Group 4 showed a significant increase in body fat when compared to group 3. Visceral fat in-group 4 showed significant increase in comparison to all other groups. TC and TG showed a significant increase in group 4 compared to all other groups. HDLc in group 4 was increased compared to group 1 and 2 but not significant when compared to group 3. Similarly, LDLc reported a significant increase (*P* <0.001) in all groups when compared to group 1 but when group 2 and group 3 were compared to group 4 no statistically significant differences were seen (Table 3).

**Table 3:** Pearson correlations of Reflection Index and Stiffness Index with age, body mass index, heart rate, systolic blood pressure, diastolic blood pressure, body fat, visceral fat and lipid profile (n=347)

**Parameters**	**Reflection index**		**Stiffness index**	
	**r**	***P*** **value**	**r**	***P*** **v alue**
Age (yr)	0.10	0.060	0.03	0.610
Body mass index	0.53	0.001	0.27	0.001
Heart rate (b/min)	0.19	0.001	0.17	0.001
Systolic blood pressure (mmHg)	0.32	0.001	0.27	0.001
Diastolic blood pressure (mmHg)	0.12	0.020	0.14	0.009
Body fat (%)	0.08	0.140	0.35	0.001
Visceral fat (%)	0.29	0.001	0.49	0.001
Total cholesterol (mg/dL)	0.31	0.001	0.58	0.001
Triglycerides (mg/dL)	0.34	0.001	0.48	0.001
High density lipoprotein cholesterol (mg/dL)	-0.02	0.750	-0.04	0.430
Low density lipoprotein cholesterol (mg/dL)	0.21	0.001	0.45	0.001

Pearson's correlation was done to assess the strength of association of various factors with RI and SI. RI was found to be positively correlated with BMI, HR, BP, VF, TC, TG, and LDLc, whereas no significant association was reported with age, BF, and HDLc. RI was found to be moderately correlated to BMI (r=0.53)(*P* <0.001). In case of SI, positive correlation was reported with BMI, HR, BP, BF, VF, TC, TG and LDLc and no association was found between age and HDLc. SI was found to be weakly correlated to BMI (r=0.27) (*P* <0.001) (Table 3).

Univariate regression analysis was carried out to assess the independent contribution of various parameters to RI and SI (Table 4). BMI, HR, SBP, DBP, VF, TC, TG and LDLc showed to have significant impact on RI and SI.

**Table 4:** Univariate regression analysis of Reflection index and Stiffness index(as dependable variables) with various other associated factors (as independent variables) in the entire group (n=347)

**Variables**	**Standardized regression** **coefficient B**	**95% CI**	***P*** **-values**
**Independent variables (Reflection index)**
Body mass index	0.27	0.39, 0.85	0.010
Heart rate	0.19	0.01, 0.34	0.001
Systolic blood pressure	0.32	0.24, 0.45	0.001
Diastolic blood pressure	0.12	0.01, 0.29	0.020
Visceral fat	0.29	0.49, 0.99	0.001
Total cholesterol	0.30	0.09, 0.18	0.001
Triglycerides	0.35	0.11, 0.19	0.001
Low density lipoprotein cholesterol	0.20	0.11, 0.34	0.001
**Independent variables (Stiffness index)**
Body mass index	0.55	0.15, 0.21	0.001
Heart rate	0.17	0.01, 0.05	0.001
Systolic blood pressure	0.27	0.03, 0.06	0.001
Diastolic blood pressure	0.14	0.01, 0.05	0.010
Body Fat	0.35	0.06, 0.09	0.001
Visceral fat	0.49	0.14, 0.21	0.001
Total cholesterol	0.52	0.03, 0.04	0.001
Triglycerides	0.48	0.02, 0.04	0.001
Low density lipoprotein cholesterol	0.46	0.06, 0.08	0.001

## Discussion

The overall prevalence of prehypertension in the present study was reported as 42.94% and there was an association between higher BMI and prehypertension. In Punjab, India, prehypertension and hypertension were more prevalent among the overweight and obese subjects^14^. BMI also contributes to sympathovagal imbalance, in the form of vagal withdrawal is more pronounced among the prehypertensives^15^. Age was significantly increased among the prehypertensive group but no correlation was reported with RI and SI.

Arterial stiffness was evaluated using indices namely reflection index and stiffness index. RI measures the vascular tone of small arteries and SI measures large artery stiffness and is an indicator of pulse wave velocity (PVW). In our study, individuals, reporting prehypertension with increased BMI showed an increase in both RI and SI in comparison to prehypertensive individuals with normal BMI. Moreover, in case of normotensive individuals, subjects with higher BMI showed a significant increase in stiffness indices. This clearly depicts that increased BMI plays a significant role in causing vascular damage. Serum leptin concentrations increased in case of obesity may predict the development of arterial stiffness in coronary artery disease patients^16^ and a useful diagnostic surrogate of cardiometabolic syndrome in hypertensive patients^17^.

Total body fat (BF) and Visceral fat (VF) were significantly increased in individuals with prehypertension and higher BMI. BF showed positive correlation with SI but not with RI. VF was also found to be significantly correlated with both RI and SI. An excess visceral body fat and total percentage body fat are strongly associated with higher risk of prehypertension and hypertension^18,19^. Visceral adiposity index, an indicator of visceral fat, may be employed as a surrogate marker for the assessment of obesity and the effects of obesity on arterial stiffness^20^. The underlying mechanism that may be responsible is that an increase in the intramyocellular and extramyocellular lipids is potent risk factors for arterial stiffness^21^. Visceral fat contributes to the development of vascular stiffness by attenuating endothelial function, enhancing inflammatory cytokine production and reducing adiponectin secretion^22^.

TC, TG, HDLc, and LDLc showed a significant increase in prehypertensive group with higher BMI compared to the normotensive group with normal BMI. TC, TG, and LDLc showed significant positive correlation with RI and SI. In a community based prospective study, TGs were a predictive factor for arterial stiffness^23^. Subjects with metabolic syndrome had increased arterial stiffness independent of age and BP, indicating that they were at high risk of developing cardiovascular diseases^24^. The relationship of lipids and ratio with arterial stiffness were elucidated, in which TG/HDLc were significantly correlated with arterial stiffness^25^.

Univariate regression analysis indicated that BMI, HR, SBP, DBP, BF, VF, TC, TG, and LDLc were independent predictors of RI and SI. Based on the adjusted r^2^ value, 30% of variance in large artery stiffness was contributed by BMI. BF, VF and lipid parameters also showed significant association with the development of large artery stiffness. Besides being a potential risk marker, it increases the afterload; stressing the myocardium to work with greater effort and resulting in CVDs. In large arteries, elastin and collagen predominate while the small arteries have more amounts of smooth muscles. A possible explanation for the cause of arterial stiffness with increase in blood pressure may be the increase in angiotensin II in prehypertensives^26^known to induce collagen cross-link formation in extracellular matrix and is referred as the major material source of vascular stiffening^27^ and the reason for vascular stiffening with increase in BMI may be due to increased leptin proved to participate in the vascular remodeling and arterial stiffness associated with obesity through oxidative stress and profibrotic factors^28^.In addition, leptin is known to augment the release of the vasoconstrictor endothelin-1, primarily in endothelial cells, and in cardiomyocytes, fibroblasts, and macrophages^29^, also leading to an elevation in blood pressure.

The limitations of the study include the small sample size, gender-based analysis was not carried out and is a cross-sectional study it is unable to explain the causal relationship of prehypertension and other CV risk factors. Future longitudinal and molecular studies are necessary to explain the actual cause and pathophysiological basis of prehypertension in young individuals.

## Conclusions

The study emphasizes the evaluation of arterial stiffness in individuals with higher BMI and prehypertension, in order to understand the magnitude of vascular damage occurred in the precursor stage itself. This will further help us to plan better interventional strategies at the earliest stage before any target organ damage is initiated.

## Acknowledgements

We acknowledge the Dr. Kalaiselvan, Department of Community Medicine, Sri Manakula Vinayagar Medical College and Hospital for their valuable suggestions and comments.

## Conflict of interest statement

The authors declare that we have no conflict of interest.

## Funding

None.

## Highlights

- The prevalence of prehypertension is reported as 42.94%
- Prehypertensive with higher BMI show increased vascular stiffness compared to normotensives with normal BMI.
- A significant association was detected between BMI and vascular stiffness
- Arterial stiffness could be evaluated in prehypertensive stage itself.
